# Supplementary material for: Circulating tumour DNA dynamics predict recurrence in stage III melanoma patients receiving neoadjuvant immunotherapy
Source: J Exp Clin Cancer Res. 2024 Aug 21;43:238. doi: 10.1186/s13046-024-03153-1 (PMC11337884; doi:10.1186/s13046-024-03153-1)
Supplement: Supplementary file 1 — Supplementary Material 1 [file 13046_2024_3153_MOESM1_ESM.docx]

**Circulating tumour DNA dynamics predict recurrence in stage III melanoma patients receiving neoadjuvant immunotherapy**

Wei Yen Chan^1,2^, Jenny H Lee^1,3^, Ashleigh Stewart^1,2^, Russell J Diefenbach^1,2^, Maria Gonzalez^2^, Alexander M Menzies^2,4,5^, Christian Blank^6^, Richard A Scolyer^2,4,7,8^, Georgina V Long^2,4,5,7^, Helen Rizos^1,2^

1. Faculty of Medicine, Health and Human Sciences, Macquarie University, Sydney, NSW, Australia
2. Melanoma Institute of Australia, The University of Sydney, Sydney, NSW, Australia
3. Department of Medical Oncology, Chris O’Brien Lifehouse, Sydney, NSW, Australia
4. Faculty of Medicine and Health, The University of Sydney, Sydney, NSW, Australia
5. Department of Medical Oncology, Royal North Shore and Mater Hospitals, Sydney, NSW, Australia
6. Department of Medical Oncology, Netherlands Cancer Institute (NKI), Amsterdam, The Netherlands, Department of Medical Oncology, Leiden University Medical Centre (LUMC), Leiden, The Netherlands, Department of Hematology and Medical Oncology, University Clinic Regensburg (UKR), Regensburg, Germany
7. Charles Perkins Centre, The University of Sydney, Sydney, NSW, Australia
8. Tissue Pathology and Diagnostic Oncology, Royal Prince Alfred Hospital & NSW Health Pathology, Sydney, NSW, Australia

**Background:** Neoadjuvant therapy improves recurrence-free survival (RFS) in resectable stage III cutaneous melanoma. However, accurately predicting individual recurrence risk remains a significant challenge. We investigated circulating tumour DNA (ctDNA) as a biomarker for recurrence in measurable stage IIIB/C melanoma patients undergoing neoadjuvant immunotherapy.

**Methods:** Plasma samples were collected pre-neoadjuvant treatment, pre-surgery and/or six weeks post-surgery from 40 patients enrolled in the OpACIN-neo and PRADO clinical trials. Patients received two cycles of ipilimumab (anti-CTLA-4) and nivolumab (anti-PD-1) before surgery. Cell free DNA (cfDNA) underwent unbiased pre-amplification followed by tumour-informed mutation detection using droplet digital polymerase chain reaction (ddPCR) with the Bio-Rad QX600 PCR system.

**Results:** Pre-treatment ctDNA was detectable in 19/40 (48%) patients. Among these, 17/19 (89%) zero-converted within six weeks of surgery and none recurred. Positive ctDNA, post-surgery (N=4), irrespective of pre-treatment ctDNA status, was 100% predictive of recurrence (sensitivity 44%, specificity 100%). Furthermore, ctDNA cleared prior to surgery in 7/9 (78%) patients who did not recur, warranting further investigation into ctDNA-guided surgical management.

**Conclusion:** Post-surgery ctDNA positivity and zero-conversion are highly predictive of recurrence, offering a window for personalised modification of adjuvant therapy.

**Key words**: circulating tumour DNA, stage III melanoma, neoadjuvant therapy, recurrence risk

1. **Introduction**

Stage III melanoma is characterised by the spread of tumour to regional lymph nodes and/or subcutaneous sites and accounts for approximately 15% of new melanoma cases. Treatment for many patients with resectable stage III melanoma involves total lymph node dissection (TLND) followed by adjuvant immune checkpoint inhibitor (ICI) or targeted (BRAF/MEK inhibitor) therapy. The use of adjuvant therapy has improved recurrence-free survival (RFS) by 35-50%, but 40-50% of patients will still recur within 3-5 years of surgery [1, 2, 3]. The true rate of recurrence is likely greater as 15-20% of melanoma patients develop disease recurrence prior to initiation of adjuvant therapy, indicating the presence of micrometastatic disease at time of surgery [4].

Following the success of ICI therapy in both the adjuvant and metastatic settings, these agents have now become standard of care in the neoadjuvant setting. The phase II SWOG-1801 study (NCT03698019) demonstrated that patients with resectable stage III/IV melanoma have significantly longer event-free survival (EFS) when treated with single agent anti-PD-1 therapy before and after surgery (neoadjuvant-adjuvant treatment) compared to patients receiving adjuvant anti-PD-1 therapy alone [5]. Additionally, the phase III NADINA clinical trial (NCT04949113) which utilised neoadjuvant dual checkpoint inhibitor therapy, confirmed the superiority of neoadjuvant-adjuvant therapy over adjuvant therapy alone in improving event-free survival (12-month EFS of 83.7% versus 57.2%) [6]. Similarly, the OpACIN (NCT02437279), OpACIN-neo (NCT02977052) and PRADO (NCT02977052) clinical trials evaluating neoadjuvant ipilimumab (anti-CTLA-4) in combination with nivolumab (anti-PD-1) in stage III melanoma, demonstrated high pathologic response rates (pRR; 74-78%) [7, 8, 9]. Pathological response after neoadjuvant therapy in stage III melanoma is a good predictor of outcome, correlating with improved RFS and overall survival (OS) [10]. Patients achieving complete pathological response (pCR; 0% viable tumour cells) rarely experience recurrence (2-year RFS 89% and OS 95%), whereas patients without pCR have markedly worse outcomes (2-year RFS 50% and OS 83%) [10].

Accurately distinguishing between patients cured by surgery alone (38%), those protected from recurrence by adjuvant systemic therapy (~10%), and those who will experience recurrence despite both treatment modalities (55%) remains rudimentary [1]. Although pathological response is a valuable and useful measure to stratify recurrence risk, predicting individual risk of recurrence is an ongoing challenge, particularly for the 35% of stage III patients with pathological non-response (pNR; >50% viable tumour cells). This group of patients exhibit varied outcomes, with 50% of patients experiencing early recurrence (within 12 months post-surgery) [10]. Hence, there is an urgent need for more accurate biomarkers to predict outcomes and tailor the treatment of resectable stage III melanoma patients accordingly.

Analysis of circulating tumour DNA (ctDNA) in liquid biopsies allows for the dynamic molecular and biological characterisation of disease in real-time and is easily repeatable. However, the use of ctDNA for minimal residual disease (MRD) detection is challenging due to the minute fraction (often less than 0.01%) of tumour DNA within the total pool of cell free DNA (cfDNA) [11]. In this study, we optimised tumour-informed ctDNA detection to investigate its predictive value in stage III melanoma patients receiving curative intent neoadjuvant immunotherapy and surgery. Here, we demonstrate that ctDNA changes pre-treatment and post-surgery (i.e. ctDNA clearance versus persistence) is a sensitive and specific biomarker of disease recurrence which could significantly impact clinical decision making.

1. **Methods and Materials**
   1. *Patients and treatment*

A total of 40 patients with resectable stage IIIB-D nodal melanoma receiving neoadjuvant ipilimumab and nivolumab as part of the phase II OpACIN-neo (N=20) clinical trial and the Phase II PRADO expansion cohort (N=20) were included in this study [7, 9, 12]. All patient samples were obtained from a single institution, the Melanoma Institute of Australia, where 72 patients were enrolled (PRADO, N=34; OpACIN-neo, N=38). Among these 72 patients, 40 patients with a driver mutation and a commercially available ddPCR primer/probe assay were selected. All patients in OpACIN-neo underwent index node resection followed by TLND, whereas patients in PRADO had index lymph node resection only followed by pathological response-driven therapy [7, 9]. Excluding two patients who experienced delayed surgery due to an immune-related adverse event (grade 3 and 4 colitis) after receiving a single cycle of ICI, the timing of surgery following the last dose of neoadjuvant immunotherapy ranged from 1.7 to 8 weeks (median: 3.2 weeks in 38 patients). Pathological response was evaluated on the resection specimen and defined as pathological complete response (pCR; 0% viable tumour cells), near pCR (<10% viable tumour cells), pathological partial response (pPR; >10% to ≤50% viable tumour cells) and pathological non-response (pNR; >50% viable tumour cells). Patients in PRADO with pCR and near pCR (defined as major pathological response; MPR) underwent surveillance, patients with pPR had subsequent TLND without adjuvant therapy, and patients with pNR underwent TLND plus adjuvant systemic treatment (nivolumab or dabrafenib and trametinib) for 52 weeks with or without local radiotherapy [9]. All patients had a follow up CT scan every 12 weeks as part of surveillance [9].

Patient demographics and clinicopathological parameters including American Joint Committee on Cancer (AJCC) stage, number of tumour-involved lymph nodes (in the TLND specimen), maximum dimension of the largest melanoma deposit (in the TLND specimen), presence of extranodal extension (ENE), baseline serum lactate dehydrogenase (LDH), and maximum positron emission tomography (PET) standardised uptake value (SUV) were included in the analysis. Written consent was obtained from all patients and research complied with ethical regulation (Human Research ethics approval from Royal Prince Alfred Hospital Protocol No X15-0454 & 2019/ETH06874 and Protocol No X15-0311 & 2019/ETH06854). Plasma samples from consented healthy donors were acquired and analysed under Macquarie University Human Research ethics, Protocol No 2793.

- 1. *Cell free DNA (cfDNA) analysis*

Tumour mutation-informed ctDNA analyses was carried out on patient plasma samples collected prior to commencement of neoadjuvant immunotherapy (pre-treatment; median 1.0 days; range 0.0 to 8.0 days) and within six weeks of index lymph node resection (post-surgery; median 5.9 weeks; range 0.0 to 7.3 weeks). A single driver mutation was identified for each primary tumour tissue from whole exome sequencing data. Commercially available primer/probe assays were used in all cfDNA analyses (BioRad Laboratories, CA; Supplementary Table 1). Plasma samples (median volume 3.5 ml; range 3.0 to 5.0 ml) were processed as previously described [13] except that DNA was eluted in 100µl of ultrapure distilled water. ctDNA was amplified in 22µl reactions using the BioRad QX600 AutoDG ddPCR system and only PCR reactions with more than 10,000 droplets were accepted for downstream analysis.

The DNA TOP-PCR cfDNA pre-amplification kit (Top Science Biotechnologies, Taiwan, Cat No. D01) was used to enhance ctDNA sensitivity [14]. Ligation and amplification were performed as described by the manufacturer, except that input cfDNA was increased to 20ng cfDNA and amplified for only 5 cycles. The TOP-PCR pre-amplification method was implemented for pre-treatment cfDNA samples with low/undetectable ctDNA (i.e. less than 10 ctDNA droplets detected via standard ddPCR), and for all post-surgery cfDNA samples. For pre-amplification experiments, 5µl of pre-amplified cfDNA (10-40ng) underwent three independent ddPCR experiments to validate ctDNA positivity.

ddPCR specificity thresholds were derived from negative control PCR reactions using DNA derived from healthy individuals and neonatal human dermal fibroblasts (HDF1314, Cell Applications, San Diego, CA). Control ddPCR reactions were performed with and without pre-amplification, with an average of 15 ddPCR control runs per probe. Positive controls for ddPCR reactions using gblock mutant DNA (Integrated DNA Technologies, IA) were included in each ddPCR run. TOP-PCR amplified post-surgery samples were considered positive only when two independent ddPCR experiments each showed at least two droplets positive for ctDNA (FAM^+^/HEX^-^). To examine dynamic ctDNA changes from pre-treatment to post-surgery (i.e. zero conversion), the pre-treatment sample was required to have at least five ctDNA droplets (FAM^+^/HEX^-^) to be considered positive.

*Statistical analysis*

Patient and disease characteristics were summarised according to pre-treatment ctDNA detectability (i.e five ctDNA droplets (FAM^+^/HEX^-^)). Frequencies and percentages according to ctDNA detectability with their corresponding P-values were calculated using Fisher’s exact test. RFS and melanoma-specific survival (MSS) were defined as time from surgery until date of first recurrence (local, locoregional or distant metastasis) or death from melanoma, respectively. RFS and MSS were calculated using the Kaplan–Meier method, and the log-rank test was used for comparison of survival. Hazard ratio estimates were based on the log-rank test. Follow-up duration was calculated from date of commencement of neoadjuvant therapy until date of death from melanoma or loss to follow-up or census date of 19 July 2024. Descriptive statistics was carried out using *jamovi* (version 2.3.28) and all other analyses were carried out using GraphPad Prism (version 10.0.3).

1. **Results**
   1. *Patients and treatment*

This study included 40 patients with stage IIIB/C melanoma enrolled in the OPACIN-neo or PRADO trials at Melanoma Institute Australia. Most patients (34/40; 85%) received two cycles of neoadjuvant ICI with six patients receiving only one ICI cycle due to severe immune-related adverse events. Mean cohort age was 57 years (range 19 to 85), 25/40 (63%) patients were male, and 16/40 (40%) had a *BRAF* mutation (Supplementary Figure 1). Pre-treatment ctDNA was detectable (with pre-amplification) in 19/40 (48%) patients. Patient demographics and clinicopathological features, stratified according to pre-treatment ctDNA status are summarised in Table 1.

**Table 1**. Clinicopathological characteristics and neoadjuvant treatment outcomes of the study population stratified according to undetectable or detectable pre-treatment ctDNA.

| Characteristics | Total N = 40 (%) | Pre-treatment Undetectable ctDNA, N=21 (%) | Pre-treatment Detectable ctDNA, N=19 (%) | P-value^a^ |
| --- | --- | --- | --- | --- |
| Age in Years, Range (Mean) | 19-85 (57) | 19-85 (55) | 32-74 (59) |  |
| Sex, n (%)  Male  Female | 25 (62.5)  15 (37.5) | 14 (66.7)  7 (33.3) | 11 (57.9)  8 (42.1) | 0.7451 |
| ECOG PS, n (%)  0  1 | 37 (92.5)  3 (7.5) | 19 (90.5)  2 (9.5) | 18 (94.7)  1 (5.3) | 1.0000 |
| Primary Site, n (%)  Upper/Lower Limb  Chest/Abdomen/Back  Head/Neck  Occult^b^ | 14 (35.0)  12 (30.0)  5 (12.5)  9 (22.5) | 9 (42.9)  5 (23.8)  3 (14.3)  4 (19.0) | 5 (26.3)  7 (36.9)  2 (10.5)  5 (26.3) | 0.7062 |
| Primary Tumour Breslow, n (%)  ≤2.0mm  >2-4.0mm  >4.0mm  Occult^b^  Unknown^b^ | 14 (35.0)  5 (12.5)  5 (12.5)  9 (22.5)  7 (17.5) | 10 (47.7)  2 (9.5)  4 (19.0)  4 (19.0)  1 (4.8) | 4 (21.1)  3 (15.8)  1 (5.3)  5 (26.3)  6 (31.6) | 0.0937 |
| Primary Tumour Ulceration, n (%)  Absent  Present  N/A (Occult primary)^b^  Unknown^b^ | 17 (42.5)  5 (12.5)  9 (22.5)  9 (22.5) | 13 (61.9)  3 (14.3)  4 (19.0)  1 (4.8) | 4 (21.1)  2 (10.5)  5 (26.3)  8 (42.1) | 0.3022 |
| Mutation Status, n (%)  BRAF^c^  NRAS^d^  Non-BRAF (TP53/TERT) | 20 (50.0)  15 (37.5)  5 (12.5) | 11 (52.4)  7 (33.3)  3 (14.3) | 9 (47.4)  8 (42.1)  2 (10.5) | 0.9094 |
| Disease Stage (AJCC 8^th^ ed), n (%)  IIIB  IIIC | 23 (57.5)  17 (42.5) | 13 (61.9)  8 (38.1) | 10 (52.6)  9 (47.4) | 0.7496 |
| Nodal status (clinical), n (%)  N1b  N2b  N3b, N3c | 26 (65.0)  7 (17.5)  7 (17.5) | 14 (66.7)  4 (19.0)  3 (14.3) | 12 (63.2)  3 (15.7)  4 (21.1) | 0.9030 |
| Number of Tumour-Involved Lymph Nodes (Pathological), n (%)  0  1-2  ≥3 | 10 (25.0)  23 (57.5)  7 (17.5) | 6 (28.6)  11 (52.4)  4 (19.0) | 4 (21.1)  12 (63.2)  3 (15.7) | 0.8323 |
| Size Of Largest Melanoma Deposit, n (%)  <35mm  ≥35mm | 23 (57.5)  17 (42.5) | 16 (76.2)  5 (23.8) | 7 (36.8)  12 (63.2) | 0.0238 |
| Extranodal Extension, n (%)  No  Yes | 31 (77.5)  9 (22.5) | 13 (61.9)  8 (38.1) | 18 (94.7)  1 (5.3) | 0.0214 |
| Lactate Dehydrogenase^e^, n (%)  ≤ULN  ≥ULN  Not measured^b^ | 33 (82.5)  5 (12.5)  2 (5.0) | 19 (90.5)  2 (9.5)  0 (0.0) | 14 (73.7)  3 (15.8)  2 (10.5) | 0.6396 |
| PET SUV Max, n (%)  Low (<5)  Moderate (5-10)  Intense (10-15)  Very Intense (>15)  Not available^b^ | 2 (5.0)  9 (22.5)  8 (20.0)  17 (42.5)  4 (10.0) | 1 (4.8)  7 (33.3)  4 (19.0)  8 (38.0)  1 (4.8) | 1 (5.3)  2 (10.5)  4 (21.0)  9 (47.4)  3 (15.8) | 0.5155 |
| Best RECIST Response, n (%)  Complete response (CR)  Partial response (PR)  Stable disease (SD)  Progressive disease (PD) | 3 (5.2)  20 (51.2)  14 (35.9)  3 (7.7) | 2 (9.5)  7 (33.3)  10 (47.7)  2 (9.5) | 1 (5.3)  13 (68.4)  4 (21.0)  1 (5.3) | 0.0972 |
| Pathological Response, n (%)  Complete Response (pCR)  Near pCR  Partial Response (pPR)  Non-Response (pNR) | 17 (42.5)  5 (12.5)  4 (10.0)  14 (35.0) | 8 (38.1)  0 (0.0)  2 (9.5)  11 (52.4) | 9 (47.4)  5 26.3)  2 (10.5)  3 (15.8) | 0.0153 |

^a^Fisher’s exact probability test was used to assess the relationship between detectable and undetectable pre-treatment ctDNA and the indicated features.

^b^Not included when calculating P-value

^c^*BRAF* mutations included *BRAF*^V600E^, *BRAF*^V600K^, *BRAF*^K601E^

^d^NRAS mutations include *NRAS^Q61R^, NRAS^Q61L^, NRAS^Q61K^*

^e^Lactate Dehydrogenase upper limit of normal used was 233u/L

ctDNA, circulating tumour DNA; ECOG, eastern cooperative oncology group; BMI, body mass index; ULN, upper limit of normal; PET, positron emission tomography.

- 1. *Pre-treatment ctDNA status*

The average cfDNA yield prior to neoadjuvant therapy was 10.2 ng/mL plasma (range 5.4 – 27.3 ng/mL plasma). Pre-treatment ctDNA was detectable in 12/40 (30%, at least 5 ctDNA^+^ droplets) and 19/40 (48%, at least 5 ctDNA^+^ droplets) patients without or with cfDNA pre-amplification, respectively. After pre-amplification, pre-treatment ctDNA was detectable in 74% (14/19) of patients with MPR, 10% (2/19) of pPR patients and 16% (3/19) of pNR patients. Pre-treatment ctDNA detectability was not predictive of recurrence (p-value = 0.1328). The distribution of mutations between detectable and undetectable ctDNA was balanced and not influenced by the pre-amplification step (Supplementary Figure 2, Fisher’s exact test p-value = 0.2538).

There was a significant association between detectable pre-treatment ctDNA and the absence of extranodal extension (p-value = 0.0214), larger size of melanoma deposit (p-value = 0.0238), and favourable pathological response (p-value = 0.0153) (Table 1). In this cohort there was no evidence of associations between detectable ctDNA pre-neoadjuvant therapy and disease stage (p-value = 0.7496), clinical nodal status (p-value = 0.9030), LDH levels (p-value = 0.6396), radiological RECIST response (p-value = 0.0972) or PET SUV max (p-value = 0.5155) (Table 1).

*3.3 Patient response to neoadjuvant ICI*

Major pathological responses (combined pCR and near pCR) were seen in 22/40 (55%) patients, including 17 (43%) with pCR and 5 (22%) with near pCR. Partial pathological response (pPR) was observed in 4/40 (10%) patients and pNR in 14/40 (35%) patients (Table 1). Among the 14 patients with pNR, seven received adjuvant therapy; only four completed the 12-month adjuvant treatment course, and three discontinued early due to toxicity (two on BRAF/MEK inhibitor therapy, and one on anti-PD-1 monotherapy). Notably, all four *BRAF* mutant pNR patients who received adjuvant therapy were treated with targeted BRAF/MEK inhibitor treatment after neoadjuvant immunotherapy. Of the remaining seven pNR patients that did not receive adjuvant systemic therapy; six patients in OpACIN-neo proceeded to surveillance as per trial protocol, and one had grade 3 toxicity whilst undergoing neoadjuvant therapy (only receiving one cycle pre-surgery).

After a median follow-up of 54 months (4.5 years; range 6 to 76 months) from commencement of neoadjuvant therapy, 9/40 (23%) patients (eight pNR and one pPR) had recurred with a median time to recurrence of 8 months (range 1 to 28 months). Two patients recurred locoregionally, one had locoregional followed by distant recurrence and six patients had distant recurrence (Figure 1).


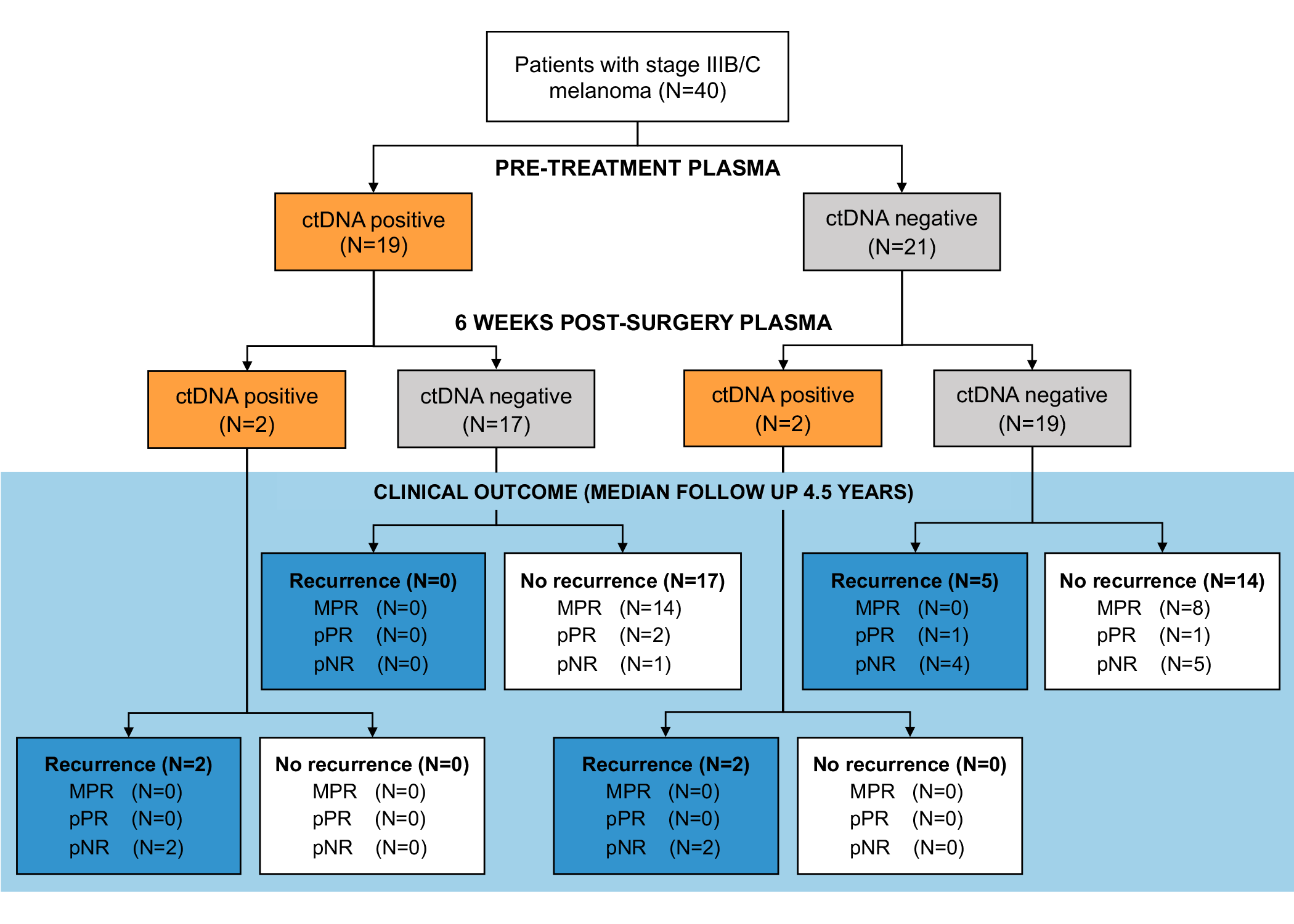


**Figure 1**. **ctDNA detectability and recurrence in high-risk stage III melanoma patients treated with neoadjuvant immunotherapy**

Flow chart showing pre-treatment and post-surgery plasma ctDNA detection and clinical recurrence. MPR, major pathological response; pPR, pathological partial response; pNR, pathological non-response.

*3.4 Dynamic peri-operative ctDNA changes predict recurrence*

The predictive value of ctDNA was especially evident in the 19 patients with detectable pre-treatment ctDNA. In this subgroup, ctDNA became undetectable (i.e. zero converted) in 17/19 (89%) patients by six weeks post-surgery, and none of these patients (0/17) experienced recurrence (Figure 2). Most patients who achieved ctDNA clearance had a MPR (14/17) however, it is noteworthy that three patients cleared their ctDNA despite not achieving a MPR to neoadjuvant therapy (Supplementary Table 2). The remaining two patients (2/19; 11%) with persistently detectable ctDNA after surgery had pNR and both (2/2; 100%) experienced early recurrence at eight months post-surgery (Supplementary Figure 3). Thus, when pre-treatment ctDNA was detectable, the sensitivity and specificity of ctDNA post-surgery in predicting recurrence were both 1.000 (95% CI: 0.1777 – 1.000 and 0.8157 – 1.000, respectively).


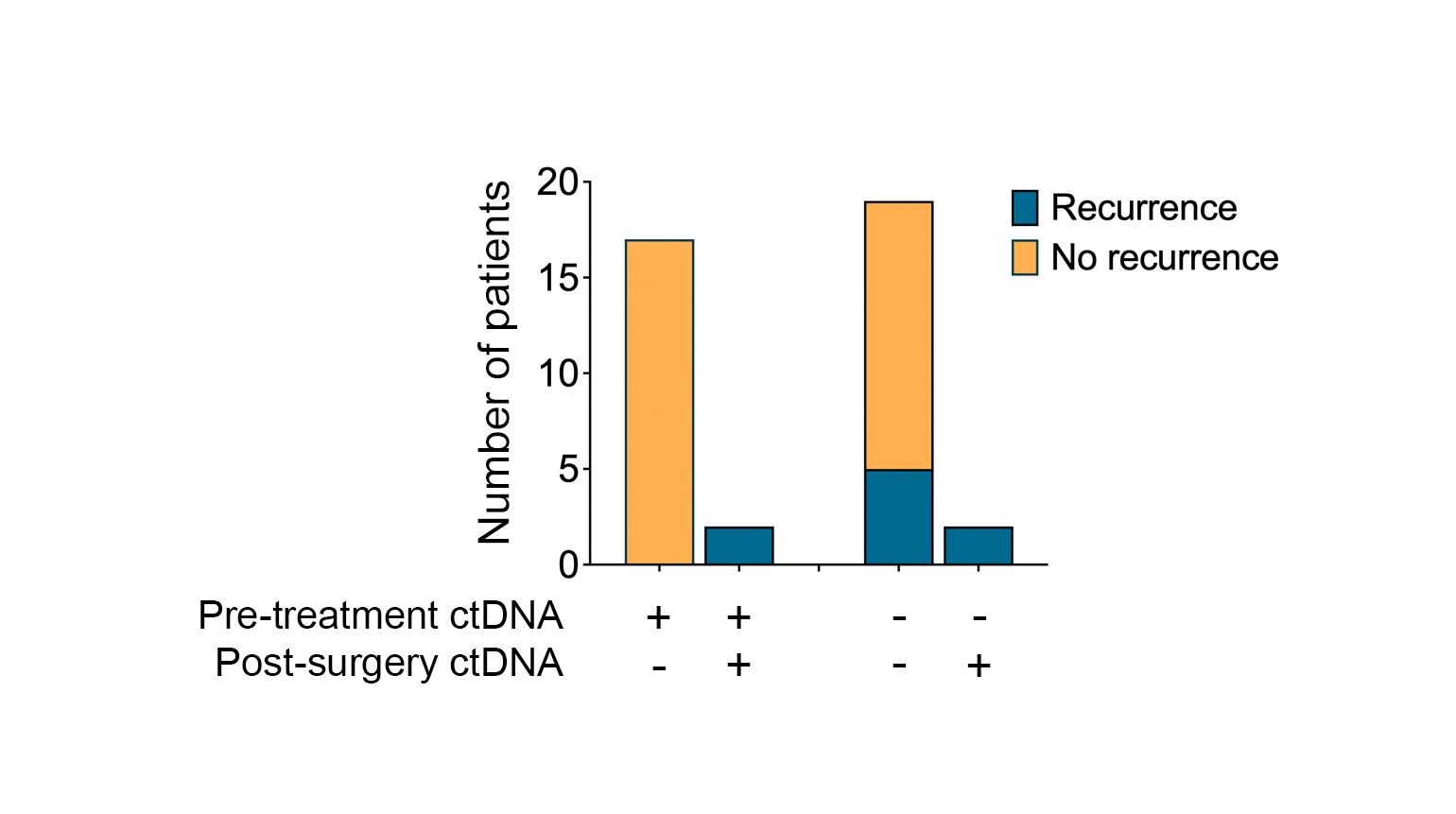


**Figure 2. ctDNA zero-conversion predicts recurrence**

Bar graph shows the recurrence status of patients according to pre-treatment and post-surgery ctDNA status.

Among the 21 patients with undetectable pre-treatment ctDNA, the post-surgical ctDNA sample (collected six weeks following surgery) was only valuable if it became detectable. Specifically, two (2/21; 9.5%) patients had ctDNA that transitioned from undetectable pre-treatment to detectable post-surgery and both patients recurred (Figure 2). These patients had a pNR and developed distant disease recurrence (at one month and 28 months post-surgery). Importantly, all patients with positive ctDNA post-surgery (4/4; 100%), regardless of pre-treatment ctDNA status, recurred.

ctDNA was of limited benefit in patients with consistently undetectable ctDNA at pre-treatment and post-surgery. Among these 19 patients, fourteen (14/19; 74%) remained recurrence-free, and five (5/19; 26%) experienced recurrence (Figure 2). It is noteworthy that of these five patients who recurred despite a negative ctDNA post-surgery, time to recurrence spanned 8 to 22 months and two patients received adjuvant therapy. The pattern of recurrence varied with two patients recurring locoregionally (managed with surgical resection), one recurred locoregionally (resected) followed by late distant recurrence (extracranial), and two recurred within 12 months with distant metastasis (predominantly intracranial) (Supplementary Figure 3). Thus, in patients with undetectable pre-treatment ctDNA, the sensitivity of ctDNA post-surgery was only 0.2857 (95% CI: 0.0508 – 0.411) and specificity was 1.000 (95% CI: 0.7847 – 1.000).

In summary, positive ctDNA post-surgery accurately predicts patients who will recur (100% positive predictive value), while zero-conversion of ctDNA post-surgery identifies patients who will not recur (sensitivity and specificity of 100% for recurrence if pre-treatment ctDNA is detectable). For the whole cohort, the post-surgery ctDNA assay demonstrated a sensitivity of 44% (test was positive in 4 out of 9 patients that recurred) and specificity of 100% (all 21 patients that did not experience recurrence tested negative for ctDNA). Median RFS was 8.0 months in patients with detectable ctDNA post-surgery compared with median not reached in patients with undetectable ctDNA [HR 10.8 (95% CI 0.8-142.4); p<0.0001] (Figure 3). Furthermore, median MSS was 43.0 months for patients who were positive for ctDNA post-surgery compared with median not reached (approximately 4.5 year follow up) for those with undetectable ctDNA [HR 8.0 (95% CI 0.4-182.0); p=0.0124] (Figure 3).


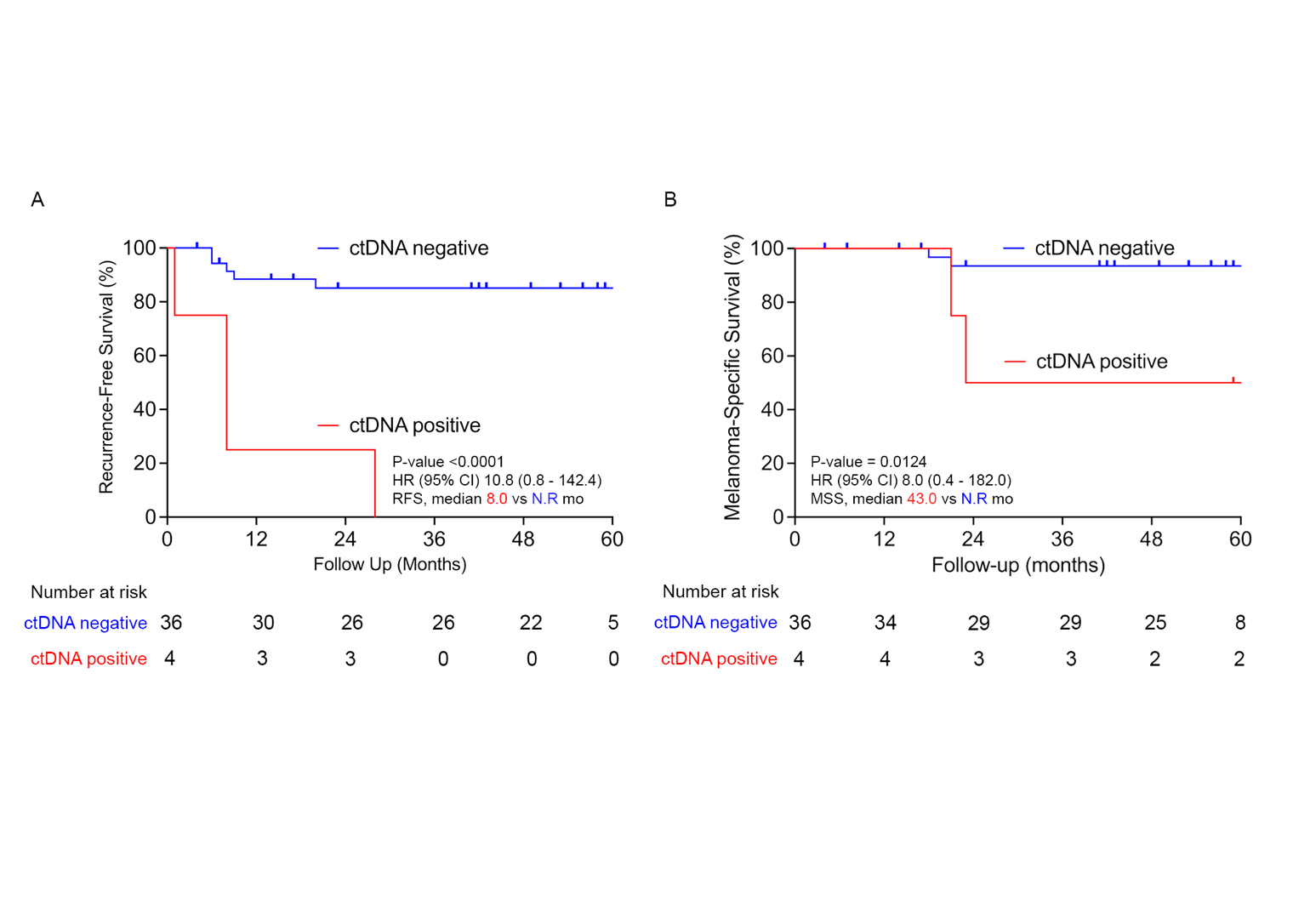


**Figure 3.** **Kaplan-Meier estimates in stage IIIB/C melanoma patients, post-neoadjuvant immunotherapy and surgery**

(A) Recurrence-free survival and (B) Melanoma-specific survival comparing patients who were ctDNA positive (red) and ctDNA negative (blue) post-surgery. Median RFS was 8.0 months in patients with positive ctDNA compared with not reached in those with negative ctDNA post-surgery. Median MSS was 43.0 months in patients with positive ctDNA compared with not reached in those with negative ctDNA post-surgery.

- 1. *Pre-surgical ctDNA for informed treatment decisions*

We next evaluated the predictive value of ctDNA collected prior to surgery. Pre-surgical plasma samples (collected 3 to 12 days prior to surgery) were collected from nine patients who did not recur (three pCR, three near pCR, two pPR and one pNR) and showed ctDNA clearance post-surgery. Of these nine patients, seven (78%) also tested negative for ctDNA after neoadjuvant therapy and prior to the scheduled surgery (Supplementary Figure 4). These findings indicate that ctDNA clearance commonly precedes surgical resection in patients who will not recur after receiving neoadjuvant combination immunotherapy.

1. **Discussion**

In this study, we evaluated the predictive utility of ctDNA for clinical recurrence in high-risk stage III melanoma patients treated with neoadjuvant immunotherapy. Importantly, our findings demonstrate that detectable ctDNA post-surgery was 100% predictive of recurrence, irrespective of the pre-treatment ctDNA status (sensitivity 44%, specificity 100%). Our results confirm that zero-conversion of ctDNA post-surgery accurately identifies patients who will not recur after undergoing neoadjuvant ICI and surgery (sensitivity and specificity of ctDNA post-surgery were both 100% when pre-treatment ctDNA was detectable). Clearance of ctDNA was also observed before surgical resection in 7/9 (78%) patients who did not recur and had detectable pre-treatment ctDNA. For these patients, including one pNR, extensive TLND may be unnecessary and further research is warranted.

Conversely, patients with negative ctDNA pre-treatment and post-surgery, retain a risk of recurrence. This subgroup of patients appears less likely to develop detectable ctDNA which may reflect specific clinical and tumour factors associated with ctDNA levels, including, age, weight, diabetes and a hypoxic tumour microenvironment [15, 16]. In this subgroup, caution is recommended in adopting de-escalation strategies solely based on ctDNA negativity.

The association of pre-treatment ctDNA with clinicopathological features, such as disease stage, primary tumour Breslow thickness, ulceration and extent of lymph node involvement has not been consistent in stage III melanoma patients [17, 18, 19]. The lack of association with many of these post-treatment features in our study is likely a result of neoadjuvant immunotherapy, which was administered to all individuals.

The strength of our retrospective study lies in the availability of prospectively collected plasma samples and granular clinical trial data, extended follow-up, and the utilisation of advanced techniques capable of detecting ultra-low levels of ctDNA in early-stage disease. Employing a pre-amplification step increased the detection rate of pre-treatment ctDNA from 30% to 48%. The potential for detecting false-positive PCR errors was mitigated through the implementation of stringent criteria for positive ctDNA detection, performing multiple independent ddPCR runs, and incorporating negative and positive controls in every analysis. Screening additional tumour-informed mutations may also improve the sensitivity of ctDNA detection in early-stage disease.

1. **Conclusion**

The clinical relevance of post-surgery ctDNA in detecting MRD holds substantial promise in patient care. We demonstrated that post-surgery ctDNA positivity was 100% predictive of recurrence, thus adding significant predictive value to pathological response. Furthermore, we show that longitudinal analysis of ctDNA dynamics, particularly in patients who zero-converted from positive ctDNA pre-neoadjuvant therapy to negative post-surgery, identifies patients who will not recur. This is particularly useful in patients without pCR, who would otherwise be subject to adjuvant systemic therapy and intense surveillance whereas treatment de-escalation is warranted based on ctDNA results. Prospective studies in the neoadjuvant setting are essential to confirm the value of ctDNA for guiding treatment and surgical decisions in melanoma patients treated with neoadjuvant ICIs.

**Declarations**

**Ethics approval and consent to participate**

Written consent was obtained from all patients and research complied with ethical regulation (Human Research ethics approval from Royal Prince Alfred Hospital Protocol No X15-0454 & 2019/ETH06874 and Protocol No X15-0311 & 2019/ETH06854). Plasma samples from consented healthy donors were acquired and analysed under Macquarie University Protocol No 2793.

**Consent for publication**

Not applicable

**Availability of data and material**

All data is available within the Article, Supplementary Information or available from the authors upon reasonable request.

**Competing Interests**

JL received honorarium from MSD, BMS, Sanofi, Roche, AstraZeneca, Novartis and sits of advisory boards for MSD, BMS and Sanofi. AMM has served on advisory boards for BMS, MSD, Novartis, Roche, Pierre-Fabre and QBiotics. RAS has received fees for professional services from SkylineDx BV, IO Biotech ApS, MetaOptima Technology Inc., F. Hoffmann-La Roche Ltd, Evaxion, Provectus Biopharmaceuticals Australia, Qbiotics, Novartis, Merck Sharp & Dohme, NeraCare, AMGEN Inc., Bristol-Myers Squibb, Myriad Genetics, GlaxoSmithKline. GVL is consultant advisor for Agenus, Amgen, Array Biopharma, AstraZeneca, Bayer, BioNTech, Boehringer Ingelheim, Bristol Myers Squibb, Evaxion, Hexal AG (Sandoz Company), Highlight Therapeutics S.L., IOBiotech, Immunocore, Innovent Biologics USA, Merck Sharpe & Dohme, Novartis, PHMR Ltd, Pierre Fabre, Regeneron, Scancell, SkylineDX B.V. All remaining authors have Declaraed no conflicts of interest.

**Funding**

This work was supported by the CLEARbridge Foundation, Melanoma Institute Australia PhD scholarship and Tour De Cure Postgraduate Research Grant. AMM is supported by a NHRMC Investigator Grant, Nicholas and Helen Moore, and Melanoma Institute Australia. RAS and JHL are supported by a National Health and Medical Research Council of Australia (NHMRC) Investigator Grant. GVL is supported by an NHMRC investigators Grant and the University of Sydney Medical Foundation.

**Authors’ contributions**

Conceived and designed study: WYC, JHL, RJD, CB, GVL, HR; Data analysis: WYC, HR, JL, RJD, MG, AS, AMM, GVL, RAS; Writing original draft: WYC, HR; Writing, reviewing and editing: all authors. All authors read and approved the final manuscript.

**Acknowledgements**

We thank the patients for donating the samples, the Melanoma Institute Australia biobank and the Macquarie University Cancer Biobank.

**References**

1. Eggermont AMM, Kicinski M, Blank CU, Mandala M, Long GV, Atkinson V, Dalle S, Haydon A, Meshcheryakov A, Khattak A, et al. Five-Year Analysis of Adjuvant Pembrolizumab or Placebo in Stage III Melanoma. NEJM Evidence. 2022;1(11).

2. Dummer R, Hauschild A, Santinami M, Atkinson V, Mandalà M, Kirkwood JM, Chiarion Sileni V, Larkin J, Nyakas M, Dutriaux C, et al. Five-Year Analysis of Adjuvant Dabrafenib plus Trametinib in Stage III Melanoma. New England Journal of Medicine. 2020;383(12):1139-48.

3. Eggermont AMM, Chiarion-Sileni V, Grob J-J, Dummer R, Wolchok JD, Schmidt H, Hamid O, Robert C, Ascierto PA, Richards JM, et al. Adjuvant ipilimumab versus placebo after complete resection of stage III melanoma: long-term follow-up results of the European Organisation for Research and Treatment of Cancer 18071 double-blind phase 3 randomised trial. European Journal of Cancer. 2019;119:1-10.

4. Weber J, Mandala M, Del Vecchio M, Gogas HJ, Arance AM, Cowey CL, Dalle S, Schenker M, Chiarion-Sileni V, Marquez-Rodas I, et al. Adjuvant Nivolumab versus Ipilimumab in Resected Stage III or IV Melanoma. New England Journal of Medicine. 2017;377(19):1824-35.

5. Patel SP, Othus M, Chen Y, Wright GP, Yost KJ, Hyngstrom JR, Hu-Lieskovan S, Lao CD, Fecher LA, Truong T-G, et al. Neoadjuvant–Adjuvant or Adjuvant-Only Pembrolizumab in Advanced Melanoma. New England Journal of Medicine. 2023;388(9):813-23.

6. Blank Christian U, Lucas Minke W, Scolyer Richard A, van de Wiel Bart A, Menzies Alexander M, Lopez-Yurda M, Hoeijmakers Lotte L, Saw Robyn PM, Lijnsvelt Judith M, Maher Nigel G, et al. Neoadjuvant Nivolumab and Ipilimumab in Resectable Stage III Melanoma. New England Journal of Medicine. 2024.

7. Rozeman EA, Hoefsmit EP, Reijers ILM, Saw RPM, Versluis JM, Krijgsman O, Dimitriadis P, Sikorska K, van de Wiel BA, Eriksson H, et al. Survival and biomarker analyses from the OpACIN-neo and OpACIN neoadjuvant immunotherapy trials in stage III melanoma. Nature Medicine. 2021;27(2):256-63.

8. Rozeman EA, Menzies AM, van Akkooi ACJ, Adhikari C, Bierman C, van de Wiel BA, Scolyer RA, Krijgsman O, Sikorska K, Eriksson H, et al. Identification of the optimal combination dosing schedule of neoadjuvant ipilimumab plus nivolumab in macroscopic stage III melanoma (OpACIN-neo): a multicentre, phase 2, randomised, controlled trial. The Lancet Oncology. 2019;20(7):948-60.

9. Reijers ILM, Menzies AM, van Akkooi ACJ, Versluis JM, van den Heuvel NMJ, Saw RPM, Pennington TE, Kapiteijn E, van der Veldt AAM, Suijkerbuijk KPM, et al. Personalized response-directed surgery and adjuvant therapy after neoadjuvant ipilimumab and nivolumab in high-risk stage III melanoma: the PRADO trial. Nature Medicine. 2022;28(6):1178-88.

10. Menzies AM, Amaria RN, Rozeman EA, Huang AC, Tetzlaff MT, van de Wiel BA, Lo S, Tarhini AA, Burton EM, Pennington TE, et al. Pathological response and survival with neoadjuvant therapy in melanoma: a pooled analysis from the International Neoadjuvant Melanoma Consortium (INMC). Nature Medicine. 2021;27(2):301-9.

11. Bohers E, Viailly P-J, Jardin F. cfDNA Sequencing: Technological Approaches and Bioinformatic Issues. Pharmaceuticals. 2021;14(6):596.

12. Keung EZ, Gershenwald JE. The eighth edition American Joint Committee on Cancer (AJCC) melanoma staging system: implications for melanoma treatment and care. Expert Review of Anticancer Therapy: Taylor and Francis Ltd; 2018. p. 775-84.

13. Lee JH, Long GV, Boyd S, Lo S, Menzies AM, Tembe V, Guminski A, Jakrot V, Scolyer RA, Mann GJ, et al. Circulating tumour DNA predicts response to anti-PD1 antibodies in metastatic melanoma. Annals of Oncology. 2017;28(5):1130-6.

14. Nai YS, Chen TH, Huang YF, Midha MK, Shiau HC, Shen CY, Chen CJ, Yu AL, Chiu KP. T Oligo-Primed Polymerase Chain Reaction (TOP-PCR): A Robust Method for the Amplification of Minute DNA Fragments in Body Fluids. Scientific Reports. 2017;7.

15. Hsiehchen D, Espinoza M, Gerber DE, Beg MS. Clinical and biological determinants of circulating tumor DNA detection and prognostication using a next-generation sequencing panel assay. Cancer Biol Ther. 2021;22(7-9):455-64.

16. Otandault A, Abraham JD, Al Amir Dache Z, Khalyfa A, Jariel-Encontre I, Forné T, Prévostel C, Chouaib S, Gozal D, Thierry AR. Hypoxia differently modulates the release of mitochondrial and nuclear DNA. Br J Cancer. 2020;122(5):715-25.

17. Lee JH, Saw RP, Thompson JF, Lo S, Spillane AJ, Shannon KF, Stretch JR, Howle J, Menzies AM, Carlino MS, et al. Pre-operative ctDNA predicts survival in high-risk stage III cutaneous melanoma patients. Ann Oncol. 2019;30(5):815-22.

18. Lee RJ, Gremel G, Marshall A, Myers KA, Fisher N, Dunn JA, Dhomen N, Corrie PG, Middleton MR, Lorigan P, et al. Circulating tumor DNA predicts survival in patients with resected high-risk stage II/III melanoma. Annals of Oncology. 2018;29(2):490-6.

19. Tan L, Sandhu S, Lee RJ, Li J, Callahan J, Ftouni S, Dhomen N, Middlehurst P, Wallace A, Raleigh J, et al. Prediction and monitoring of relapse in stage III melanoma using circulating tumor DNA. Annals of Oncology. 2019;30(5):804-14.
