# Supplementary material for: Circulating tumour DNA dynamics predict recurrence in stage III melanoma patients receiving neoadjuvant immunotherapy
Source: J Exp Clin Cancer Res. 2024 Aug 21;43:238. doi: 10.1186/s13046-024-03153-1 (PMC11337884; doi:10.1186/s13046-024-03153-1)
Supplement: Supplementary file 3 — Supplementary Material 3 [file 13046_2024_3153_MOESM3_ESM.docx]

**Supplementary Figures**


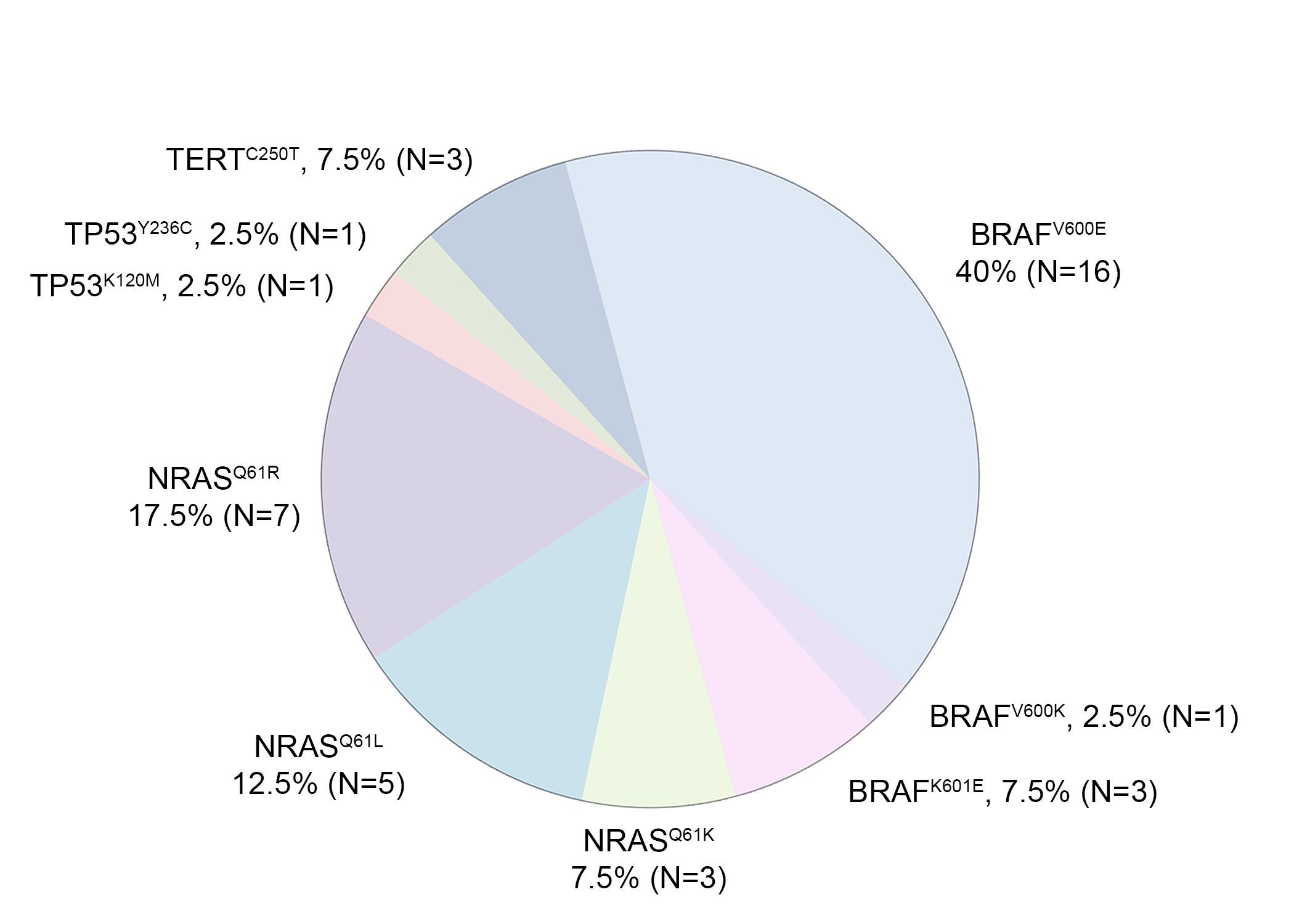


**Supplementary Figure 1.** **Frequency of tumour mutations in the melanoma cohort (N=40)**

Somatic gene mutation data obtained through sequencing of primary melanoma tissue.


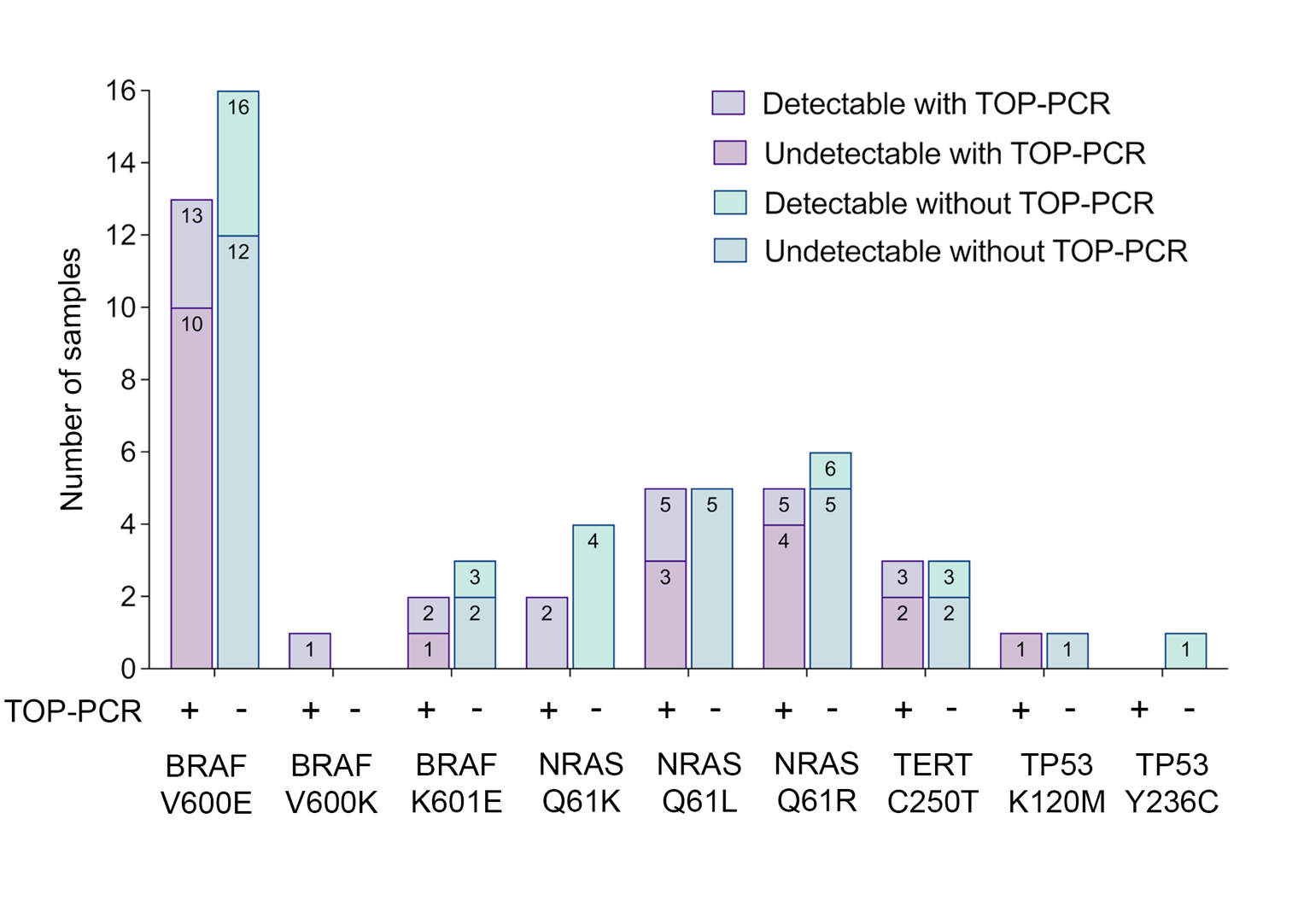


**Supplementary Figure 2.** **Association between assessed mutations and ctDNA detectability**

Distribution of tumour mutations according to ctDNA detectability at pre-treatment. One mutation was evaluated per patient sample. In total, 19 patient samples had detectable ctDNA (threshold of ≥ five mutant droplets) while 21 samples had undetectable ctDNA. Fisher’s exact test was used to evaluate the distribution of ctDNA mutations detected in unamplified and post TOP-PCR amplified patient samples (p-value = 0.2538).


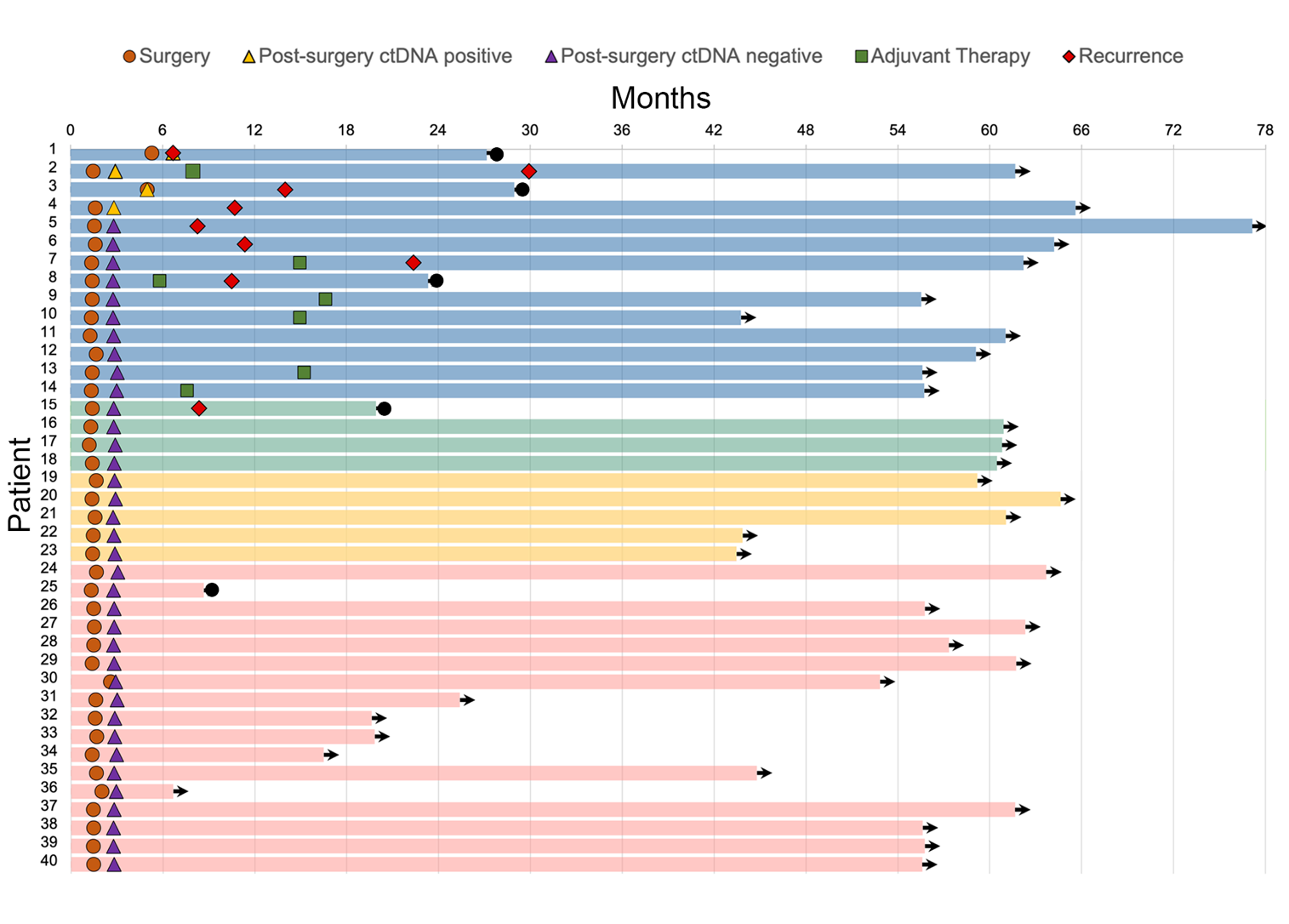


**Supplementary Figure 3. Swimmer plot of stage III melanoma patients undergoing combination neoadjuvant anti-PD-1 and anti-CTLA-4 immunotherapy**

The pathological response (blue, pathological non-response; green, pathological partial response; yellow, near pathological complete response; pink, pathological complete response) and clinical recurrence from baseline plasma collection to date of death (•) or last follow-up (→) is indicated for each patient. The longitudinal plot traces each patient’s trajectory through neoadjuvant therapy, surgery (index lymph node dissection), adjuvant therapy and recurrence events and its association with ctDNA detection post-surgery.


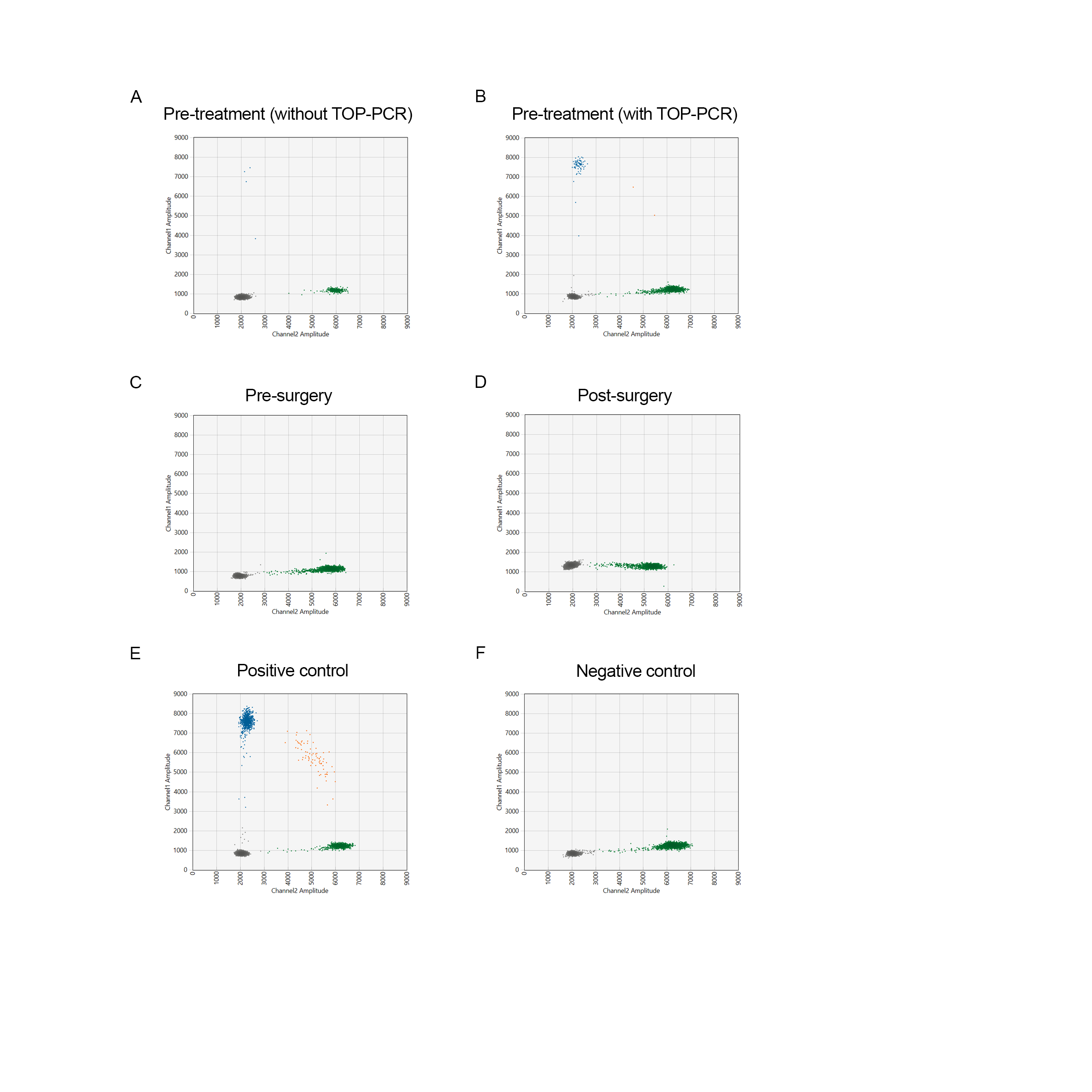


**Supplementary Figure 4.** Droplet digital PCR (ddPCR) two-dimensional (2D) plot for patient no. 24 (pathological complete responder) with NRAS p.Q61L, c.182A>T target:

A) Pre-treatment without TOP-PCR pre-amplification: 4 ctDNA (FAM^+^/HEX^-^) droplets

B) Pre-treatment with TOP-PCR pre-amplification: 72 ctDNA (FAM^+^/HEX^-^) droplets

C) Pre-surgery (week 6 plasma) with TOP-PCR pre-amplification: 0 ctDNA (FAM^+^/HEX^-^) droplets

D) Post-surgery (week 12 plasma) with TOP-PCR pre-amplification: 0 ctDNA (FAM^+^/HEX^-^) droplets

E) Positive control: NRAS Q61L positive cell line

F) Negative control: HDF1314 fibroblast cell line

**Supplementary Tables**

**Supplementary Table 1**. **Bio-Rad probes employed in ddPCR analysis**

| Assay Name | Target | Probe Fluorophore | Wet Lab Validated | Assay ID |
| --- | --- | --- | --- | --- |
| BRAF K601E | BRAF p.K601E c.1801A>G | FAM | No | dHsaIS2504434 |
| BRAF WT for K601E | BRAF WT for p.K601E c.1801A | HEX | No | dHsaIS2504435 |
| BRAF V600E | BRAF p.V600E c.1799T>A | FAM/HEX | Yes | dHsaMDV2010027 |
| BRAF V600K | BRAF p.V600K c.1798_1799GT>AA | FAM/HEX | Yes | dHsaMDV2010035 |
| NRAS Q61K | NRAS p.Q61K c.181C>A | FAM/HEX | Yes | dHsaMDV2010067 |
| NRAS Q61L | NRAS p.Q61L c.182A>T | FAM/HEX | Yes | dHsaMDV2010069 |
| NRAS Q61R | NRAS p.Q61R c.182A>G | FAM/HEX | Yes | dHsaMDV2010071 |
| TP53 K120M | TP53 p. K120M c.359A>T | FAM/HEX | No | dHsaMDS2516044 |
| TP53 Y236C | TP53 p. Y236C c.707A>G | FAM/HEX | Yes | dHsaMDV2516916 |

WT, wild type

**Supplementary Table 2. Dynamic ctDNA change relative to pathological response**

| **ctDNA detectability** | | **Pathological Response** | | | |  |
| --- | --- | --- | --- | --- | --- | --- |
| Pre-treatment | Post-treatment | pCR | near pCR | pPR | pNR | Total |
| *+* | *+* | 0 | 0 | 0 | 2 | **2** |
| *+* | *-* | 9 | 5 | 2 | 1 | **17** |
| *-* | *-* | 8 | 0 | 2 | 9 | **19** |
| *-* | *+* | 0 | 0 | 0 | 2 | **2** |
| **Total** | | **17** | **5** | **4** | **14** | **40** |

pCR, pathological complete response; pPR, pathological partial response; pNR, pathological non-response. The highlighted patients with detectable pre-treatment ctDNA that zero converted did not recur.
