# Supplementary material for: Circulating tumour DNA dynamics predict recurrence in stage III melanoma patients receiving neoadjuvant immunotherapy
Source: J Exp Clin Cancer Res. 2024 Aug 21;43:238. doi: 10.1186/s13046-024-03153-1 (PMC11337884; doi:10.1186/s13046-024-03153-1)
Supplement: Supplementary file 4 — Supplementary Material 4 [file 13046_2024_3153_MOESM4_ESM.docx]

28 July 2024

Dear Dr Castelli,

***Re: Submission of revised manuscript JECC-D-24-01408***

We thank you for the opportunity to resubmit our manuscript to the Journal of Experimental and Clinical Cancer Research.

Our manuscript has been extensively modified since the initial submission as suggested by the reviewers, and additional data and clarifications have been included.

All changes have been underlined within the manuscript.

We appreciate the thoughtful comments made by the reviewers, and the revisions suggested have strengthened the manuscript. I hope you will find our revised manuscript suitable for publication.

Please let me know if there are any additional requirements for this submission.

Yours sincerely,

**Professor Helen Rizos**

**Reviewer: 1**

1. It is therefore essential, that the authors describe the ddPCR analyses in detail. This should include:

a. the respectively used tumour mutations for this analysis. Is there any correlation between successful detection of ctDNA and the assessed mutation?

We have now included additional details on ddPCR mutation analysis. The mutations analysed were balanced across detectable and undetectable pre-treatment ctDNA, and these details are shown in the new Supplementary Figure 2 and data described on page 10, line 178-180.

b. if no commercially available assay was used, the authors should indicate the forward/reverse primers along with the probe sequences that have been used for each mutation.

This study utilised commercially available assays, with details provided in Supplementary Table 1. We have clarified these details in the manuscript (line 116-117).

c. exemplary pictures of the ddPCR scatter plots of pre-treatment / post-surgery patient versus control samples

Pictures of ddPCR scatter plots have now been added in Supplementary Figure 4.

2. Similarly, since it is a crucial step for enhancing the sensitivity of ctDNA detection, the method to perform the unbiased cfDNA pre-amplification should be outlined in more detail in the Methods and Materials part. Was a specific kit purchased from Top Science Biotechnologies?

The DNA TOP-PCR kit was purchased from Top Science Biotechnologies Inc, Taiwan (Cat #D01). The changes made to the protocol (increased input cfDNA and only 5 cycles of amplification) are described in the methods (page 5, line 122-128). A detailed technical analysis of the performance of the TOP-PCR protocol will be submitted for publication as a separate manuscript.

3. The manuscript refers to Supplementary Tables/Figures (e.g. page 5 line 115; page 6 line 143; page 12 line 218), however, no supplementary material was included or attached in the provided version of the manuscript. Yet, some information seems to be already presented in the tables/figures of the main manuscript. This should be double-checked and updated.

We apologize for the confusion. A separate supplementary document, including supplementary tables and figures was submitted with the original manuscript. We have carefully reviewed the supplementary information and ensured that all tables and figures add additional value to the manuscript.

4. page 3 lines 69: …detection is challenging due to the minute fraction (often than 0.01%) of tumour DNA… -> "often less than 0.01%"?

**Page 4, Line 73:** This sentence has been corrected as recommended (see line 73-74).

5. page 13 lines 225: … specificity of 100% (all 21 patients that tested negative for ctDNA did not experience recurrence). -> in total 26 patients tested negative for ctDNA at the post-surgery time point, however only 21 out of those 26 patients had no recurrence (Figure 1). Therefore, it should rather be the other way round: "all 21 patients that did not experience recurrence tested negative for ctDNA"

**Page 14, Line 246:** Thank you for this correction, it has now been addressed.

6. **Figure 3:** The colour coding used for discrimination between ctDNA negative (blue) and positive (red) in the graph seems to be mixed up for median RFS and MSS values. The timepoints were not reached for undetectable (blue) ctDNA, respectively.

Figure 3 has been corrected.

**Reviewer: 2**

1. Sample Size and Generalizability: The take a look at's small pattern size (30 sufferers) limits its statistical power and generalizability. Future research have to include larger cohorts to validate the findings and make sure broader applicability.

We agree that validating these findings in follow-up research is important. Significantly, we have now increased the study size to 40 stage III melanoma patients. The median follow up of these patients has also increased to 4.5 months (range 6-76 months). These additional data confirm our findings that post-surgery ctDNA positivity and zero-conversion is predictive of recurrence. We have also included an additional Supplementary Table 2, to simplify the presentation of these important findings.

2. Variability in Treatment Protocols: The variability in remedy protocols among the PRADO and OPACIN-neo trials can also introduce confounding factors. It is essential to cope with how those variations would possibly affect the outcomes and keep in mind standardizing treatment protocols in future research.

The PRADO clinical trial (phase II) is the expansion cohort of the OpACIN-Neo (phase II) trial – this detail is now included in **Page 4, Line 83**.

While we acknowledge variability in the adjuvant therapies received by some patients, all participants underwent standardised neoadjuvant combination immunotherapy (ipilimumab and nivolumab). Further, the plasma timepoints collected – pre-treatment, pre-surgery and post-surgery – were consistent across both trials. Although recurrence-free survival can be influenced by adjuvant therapy, the dynamic changes in ctDNA seen in this study remain predictive of recurrence.

3. Timing of Surgery: The huge variety in timing between the remaining dose of immunotherapy and surgical operation (1.7 to 21.4 weeks) should effect the outcomes. Standardizing the time to surgery ought to reduce variability and provide clearer insights into the results of neoadjuvant therapy.

The trial protocol standardised the time to surgery, mandating a pre-planned timepoint of six weeks from baseline, which is three weeks after the last dose of immunotherapy. There were two patient outliers who had surgery at 19 and 21 weeks respectively, after developing severe immune-related toxicity. Excluding this outlier, the timing of surgery ranged from 1.7 to 8 weeks. We have updated these details in **Page 4, line 89-92**.

4. ctDNA Detection and Analysis: The reliance on pre-amplification steps for ctDNA detection, while improving sensitivity, may additionally introduce biases or artifacts. It's vital to discuss those capacity biases and recollect alternative techniques to validate ctDNA findings.

We have included the following data to confirm the accuracy of the TOP-PCR amplification process.

a) We have included a new Supplementary Figure 2 which confirms that ctDNA detectability after TOP-PCR amplification was not associated with assessed mutation.

b) We conducted matched ctDNA detection pre- and post- amplification in 32 of 40 patients (pre-treatment sample). In 9/32 patients where ctDNA was detected without amplification, TOP-PCR confirmed all findings (i.e. it validated all 9 positive results). Additionally, TOP-PCR enhanced detection sensitivity, identifying positive ctDNA in samples that were previously negative without amplification.

c) The method we used is a commercially available (D01, Top Science Biotechnologies Inc, Taiwan), and published amplification protocol.

Please also see response 2, Reviewer #1.

5. Control Group: The absence of a manage organization that did not receive neoadjuvant immunotherapy makes it difficult to characteristic found consequences completely to the remedy regimen. Including a manipulate organization in destiny studies could provide a baseline for comparison and improve causal inferences.

It is not clear to us what the reviewer means in this instance. If the query is relating to the efficacy of neoadjuvant immunotherapy, the recently published phase III NADINA study which utilised the same neoadjuvant combination treatment (i.e. ipilimumab and nivolumab), demonstrated that neoadjuvant-adjuvant therapy is superior to adjuvant therapy alone in improving event-free survival ((12-month EFS of 83.7% versus 57.2%; **Page 3: line 50-53**). Our study focuses on ctDNA changes and its value in predicting recurrence after neoadjuvant therapy, making the inclusion of a control group less relevant in this context.

6. Association with Clinicopathological Features: The loss of affiliation among pre-remedy ctDNA detectability and clinicopathological functions, despite advanced detection strategies, warrants in addition research. Clarifying those relationships could enhance knowledge of ctDNA's position in melanoma analysis.

We acknowledge the importance of further exploring the detectability of ctDNA and its correlation with clinicopathological features. Importantly, there is a lack of clear associations between ctDNA

and clinicopathological features in other studies (as highlighted **Lines 284-286**) and the lack of association in our study may be attributed to the timing of assessing certain clinicopathological features post neoadjuvant therapy. We are committed to conducting further research in this area to gain deeper insights.

7. Retrospective Study Design: The retrospective nature of the observe limits the capability to set up causality. Prospective research are needed to verify the predictive fee of ctDNA and its potential to guide treatment decisions in real-time medical settings.

We agree that a prospective study will be important to validate the value of ctDNA in predicting recurrence as well as potentially informing the need for surgery, specifically total lymph node dissection. This was included in the conclusion (**Page 16: line 306-307**).

8. Predictive Value of Pre-remedy ctDNA: Although the take a look at highlights the predictive cost of put up-surgical ctDNA, pre-remedy ctDNA detectability was not predictive of recurrence. Further studies is wanted to explore the software of pre-remedy ctDNA in medical choice-making.

While our study reported that pre-treatment ctDNA detectability alone did not predict recurrence, this may be attributed to the influence of neoadjuvant therapy. Nevertheless, we showed that dynamic changes in ctDNA levels from pre-treatment to post-surgery can effectively identify patients at high risk of recurrence, and guide decisions regarding adjuvant therapy. This is clinically significant.

9. Longitudinal Analysis of ctDNA: The dialogue efficaciously addresses the potential of longitudinal ctDNA evaluation for figuring out patients who might not need great surgical operation or adjuvant remedy. Future research should awareness on validating those findings to support remedy de-escalation strategies.

Thank you for your comment. We believe that serial monitoring of ctDNA changes, which tracks the dynamic evolution of tumour burden, can potentially reduce the necessity of surgery, thereby sparing patients from the morbidity of a total lymph node dissection. Future prospective studies are crucial to confirm the benefits of intervening based on ctDNA status.

10. Prospective Studies and Clinical Utility: The end emphasizes the need for prospective studies to verify ctDNA's fee in guiding treatment and surgical selections. Highlighting the scientific relevance of these findings should encourage the design and implementation of such research.

We hope that with the publication of our study highlighting the importance of dynamic ctDNA changes after neoadjuvant therapy, we can inspire the design and implementation of a prospective, ctDNA-guided adjuvant trial in early-stage melanoma. Such as trial could definitively establish the benefits of this approach in terms of recurrence-free survival and overall survival and reduce the need for extensive surgery among patients. This approach will pave the way for integration of ctDNA guided strategies into routine clinical practice.
